# An Investigation of Prescription Indicators and Trends Among General Practitioners and Specialists From 2005 to 2015 in Kerman, Iran

**DOI:** 10.15171/ijhpm.2018.28

**Published:** 2018-04-25

**Authors:** Ali Masoud, Somayeh Noori Hekmat, Reza Dehnavieh, Naser Haj-Akbari, Atousa Poursheikhali, Zhaleh Abdi

**Affiliations:** ^1^Health Services Management Research Center, Institute for Futures Studies in Health, Kerman University of Medical Sciences, Kerman, Iran.; ^2^Research Center for Modeling in Health, Institute for Futures Studies in Health, Kerman University of Medical Sciences, Kerman, Iran.; ^3^Physiology Research Center, Kerman University of Medical Sciences, Kerman, Iran.; ^4^National Institute of Health Research (NIHR), Tehran University of Medical Sciences, Tehran, Iran.

**Keywords:** Prescription Indicator, Trend, General Practitioner, Specialist, Iran

## Abstract

**Background:** The World Health Organization (WHO) aims to promote strategies that ensure efficacy, safety, suitability, and cost-effectiveness of medicine prescription. Health systems should design effective mechanisms to monitor prescription and rational use of medicines at all healthcare settings. This study aimed to determine and analyze prescription patterns of general practitioners and specialists in Kerman/Iran from 2005 to 2015.

**Methods:** This is an explanatory mixed method study. Data were gathered during two phases. At the first phase, prescriptions issued by physicians during 2005-2015 were reviewed to extract information required to develop eight main prescription indicators. In the second phase, the indicators trends were presented to experts participating in expert panel to have their opinions and analyses on the data obtained in the first phase. Experts were selected based on their experience and expertise in medicine and/or health policy and/or experience in implementation of polices to promote rational use of medicines. Some experts attending the panel were a sample of physicians whose prescriptions were included in the first phase.

**Results:** Findings revealed that two indicators of the average price of prescriptions and the maximum number of medicines in each prescription had an increasing trend over the study period. Reasons including unprecedented devaluation of the Iranian Rial and willingness of young physicians to prescribe more medications were proposed as the primary contributors to the observed increasing trends. However, other indicators including types of prescribed medicines, average number of medicines per prescription, the percentage of prescriptions with more than four medications, a percentage of encounters with a corticosteroid prescribed, a percentage of encounters with an antibiotic prescribed, and a percentage of encounters with an injection prescribed decreased in the study period. Reasons of controlling initiatives adopted by the Ministry of Health, the higher responsibility of physicians, adoption of continued medical education (CME) programs, and improved knowledge of pharmacists, physicians, and patients about irrational use of medicines were proposed by participants as the main reasons for the decreasing trend.

**Conclusion:** Findings indicated that prescription indicators were better in Kerman than those of country average over the study period based on comparing the results of this study and others in Iran. However, they were non-desirable when compared to the international average. The number of factors contributes to the irrational use of medicines, including lack of knowledge among healthcare providers and patients, patients’ misunderstanding about the efficacy of some particular medicines, the high cost of drug development and manufacturing, and unavailability of effective medicines.

## Background

Establishment of an effective pharmaceutical management system is one of the most important goals of health systems worldwide.^1^ The World Health Organization (WHO) aims to promote strategies that ensure efficacy, safety, suitability, and cost-effectiveness of medicine prescription. Medicine is a strategic product of concern to all countries in such a way that a large proportion of discretionary health expenditures in both developed and developing countries allocate to procurement, distribution and consumption of medicines.^2,3^ Considering their functions, health systems are not only responsible for providing equitable access to medicines, but also they should design effective mechanisms to monitor the prescription and rational use of medicines at all settings of healthcare.^4^

WHO defined rational drug perception as “patients receive medications appropriate to their clinical needs, in doses that meet their individual requirements, for an adequate period of time, and by the lowest cost to them and their communities.”^5,6^ WHO and the International Network Rational Drug Use (INRUD) have proposed a number of indicators to assess rational use of medicines. These indicators are widely accepted as an objective standard to measure quality of medicines utilization and prescription behavior in health facilities.^5^

Inappropriate use of medicines can have adverse effects on health costs. In one hand, inappropriate use of medicines can adversely jeopardize quality of medical care and influence treatment outcomes. It can also lead to antimicrobial resistance. On the other hand, higher costs of pharmaceutical products along with technical dependency of pharmaceutical industry to developed countries may lead to either dependence on medicinal imports or permanent and temporary shortage of some types of medicines.^2,7^

Evidence shows that from 2005 to 2011, per capita consumption of tablets and capsules increased from 367 to 432 ones in Iran, indicating of a 17.7% increase in medication use. However, it seems that the best way to control irrational use of medicines is through prescription management.^8^ Studies have shown that the average number of drug per prescription among Iranian physicians is 3.07, which is higher than the standards recommended by WHO that is between 1.3 to 2. This is also true for antimicrobials and intravenous drugs, indicating that the high prescription and consumption of medicines in Iran is way higher than of recommended international standards.^9,10^ The study in one of the Iranian cities (Isfahan) showed that the administration of antibiotics and injectables in the prescriptions of physicians is common and inadequate.^11^

This study aimed to determine and analyze prescription patterns of general practitioners and specialists in Kerman province from 2005 to 2015. Iran’s Ministry of Health and Medical Education (MoHME) has developed a set of indicators including indicators developed jointly by WHO and INRUD and indicators suggested by for rational use of medicines. This set of indicators are used for this study. In order to achieve a deeper analysis of the changes in the prescription trend over that 11 years, and to better identify influential factors, quantitative results were combined by qualitative exploration of experts’ opinions.

## Methods

This is an explanatory mixed method study. Mixed method studies provide researchers with opportunities to triangulate their findings with different sources of information. As follows, two phases of data collection and analysis were undertaken:

### Gathering Quantitative Data and Analyzing Indicators Trends

During this phase, prescriptions written by physicians during 2005-2015 were reviewed to extract information required to develop the eight main indicators of (1) average price of prescriptions, (2) types of prescribed medicines, (3) average number of medicines per prescription, (4) maximum number of medicines per prescription, (5) percentage of prescriptions with more than four medicines, (6) percentage of encounters with a corticosteroid prescribed, (7) percentage of encounters with an antibiotic prescribed, and (8) percentage of encounters with an injection prescribed. These indicators are selected because the Ministry of Health has determined these indicators, collects data for them, and monitors them systematically.

Currently, prescriptions of patients received in pharmacies are electronically sent to insurance companies and then sent to the Food and Drug Administration. All electronic data are imported in the software of “Noskheh pardaz.” We have used this software data for our study indicators and investigating the trends. Data was analyzed using SPPS software version 16.

### Expert Panel and Analysis of Indicators Trends

The aim of this phase was to present the indicator’s trends to experts participating in the expert panel to have their point of view and their analysis on results obtained from the first phase. The experts were from different disciplines including health management and economics, medicine, and pharmaceutical industry. For our expert panel we selected the experts by different disciplines and specialties to investigate the issue from different aspects. For example we had general practitioner and specialist to realizing their prescription behavior. We had also experts from health management and economics, medicine, and pharmaceutical industry for realizing and considering the socio economic factors. And also we had experts from insurance as the key stakeholder and the importance of drugs supported by insurance. Experts were selected based on their experience and expertise in medicine and/or health policy and/or experience in implementation of polices to promote rational use of medicines. Table 1 shows the demographic characteristics of the participants. To create a balance between topics in group discussions, participants were selected using a proportional sampling method.^12^

**Table 1:** Location and Number of Interviewees at Each Organization

**Organization**	**Organizational Role**	**No. of Participants**
Kerman University of Medical Sciences Officials (KMO)	Deputy director for food and drug	4
	Director of think tank	
	Dean of the Faculty of Pharmacy	
	Research and Development Department of the Food and Drug Administration	
Managers and experts from the health insurance offices in Kerman province (HI)	Provincial officials and experts of health insurance	4
Healthcare provider (HR)	General practitioner (GP), specialist (SP), pharmacist (PH)	6
Researchers (R)	Experts Food and Drug Department, Researchers in the fields of Health Economics, Healthcare Management and Health Policy	6
Total		20

At the beginning of group discussions, the participants were presented the changes happened in prescriptions over the study period. Participants, then, were asked to express their views on the causes and factors influencing the changes in indicators, and to propose strategies to improve rational use of medicines in the future. Overall, two expert panels were conducted with 20 experts. All conversations were digitally recorded with the permission of participants and transcribed verbatim.

The framework analysis method consisting of ﬁve main steps (familiarization, identifying a thematic framework, indexing, charting and mapping, and interpretation) was used to analyze qualitative data. Incorporating inductive and deductive elements, this method is becoming an increasingly popular approach in health policy research.^13^

During familiarization step, a summary of the content of each interview was created. The initial thematic framework was developed by the research team members. The transcripts were checked against thematic framework through a familiarization step. Then transcribed interviews were initially indexed by one of the authors. Coding was veriﬁed by other authors and disagreements were resolved through discussion. Data were coded and categorized using MAXQDA 10 software. In charting stage, a chart was produced for each theme. All data were transferred to these charts to produce the analysis chart. Themes were written up describing the similarities and variations between participants. Finally, in the mapping and interpretation step, the charts were reviewed by all the authors to make sense of the entire data set.

## Results

**Figure 1:**
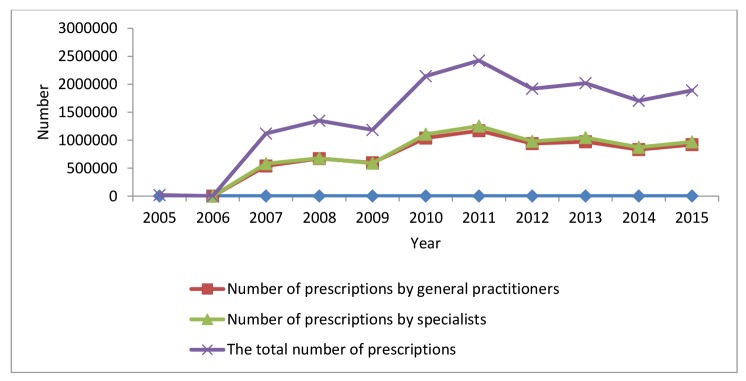
The Total Number of Drug Prescriptions by General Practitioners and Specialists (2005-2015).

The initial results showed an increase in the total number of prescriptions during those years. Table 2 displays the total number of reviewed prescriptions by general practitioners and specialists from 2005 to 2015 in Kerman. Further, the number of prescriptions by general practitioners and specialists were always equal and the increase or decrease in different years had a relatively same trend (Figure 1).

**Table 2:** Total Number of Prescriptions From 2005 to 2015

**Year**	**2005**	**2006**	**2007**	**2008**	**2009**	**2010**	**2011**	**2012**	**2013**	**2014**	**2015**
No. of prescriptions by general practitioners	**-**	484	539 559	671 903	596 789	1 039 065	1 170 248	940 367	972 848	832 887	920 842
No. of prescriptions by specialists	-	1526	581 071	677 565	588 080	1 106 817	1 253 871	980 897	1 047 210	873 081	969 821
The total No. of prescriptions	19 382	2010	1 120 630	1 349 468	1 184 869	2 145 882	2 424 119	1 921 264	2 020 058	1 705 968	1 890 663

In the current study, simultaneous gathering and analysis of data at two phases allowed the authors to identify and investigate indicators’ trends from 2005 to 2015, shown in Table 3. This matter will be explained in the below.

**Table 3:** Prescription Quality Indicators (2005-2015)

**Indicator**	**Year**											
	**2005**	**2006**	**2007**	**2008**	**2009**	**2010**	**2011**	**2012**	**2013**	**2014**	**2015**
Average price of prescriptions (IRR)	22 537	20 365	31 263	28 860	38 719	40 107	44 228	51 635	71 959	86 717	106 877
Number of prescribed medicines	342	1033	1414	1418	1726	1618	1525	1463	1305	1475	1405
Average number of medicines per prescription	3.28	3.38	3.10	3.04	3.00	2.94	2.88	2.83	2.78	2.81	2.81
Maximum number of medicines per prescription	9	12	14	13	23	14	17	14	12	13	14
Percentage of prescriptions with more than four medicines	14%	17%	13%	12%	12%	11%	10%	9%	9%	9%	10%
Percentage of encounters with a corticosteroid prescribed	0	0	0	0	17%	16%	16%	16%	15%	16%	14%
Percentage of encounters with an antibiotic prescribed	0	0	0	0	45%	44%	43%	41%	38%	40%	39%
Percentage of encounters with an injection prescribed	29%	39%	30%	30%	28%	27%	29%	28%	28%	27%	26%

### Average Price of Prescriptions

**Figure 2:**
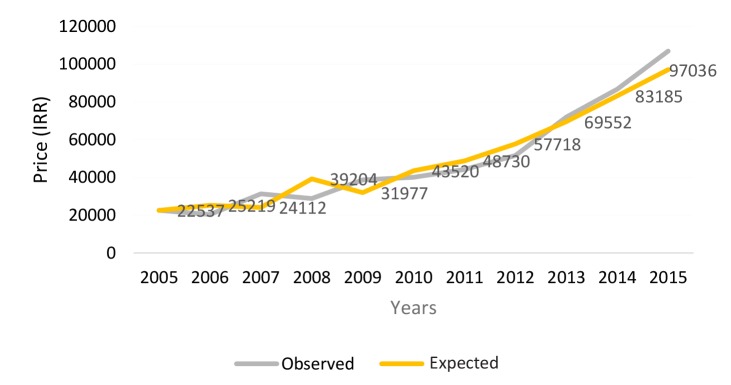
Average Price of Each Prescription.

The following figure shows that the trend of prescriptions price has risen over the 10-year period that has intensified since 2011. Experts’ analysis was that this rise was due to the influence of inflation, which affected the Iranian economy in last 10-year period: *“Since 2011, we have witnessed an increase in the price of medicines in the country due to dramatic devaluation of national currency versus US dollar…, the price of some medicines, compared to the past, have increased several folds, which has put a fiscal pressure on both the health system and people…”* (R2). To test this hypothesis, by using the inflation rates in this 10-year period, the expected prices were calculated with the annual inflation rate (which is shown by double line in Figure 2). As noted above, for most of this period years, the observed prices are close to the expected price levels. Therefore, it can be said that the main part of price changes is justified by the trend of annual inflation rate changes.

An increase in price of raw material in the pharmaceutical sector was the other reason mentioned for the increase in average price of medicines: *“The government somehow controls inflation rate in the pharmaceutical sector, but following the increase in factors such as price of raw materials, staff costs (Iranian worker’s costs), transport costs and so on, the price of medicines increased by 16 to 18% annually, a considerable increase which can be seen in the trends as well”* (KMO1).

### Types of Prescribed Medicines

**Figure 3:**
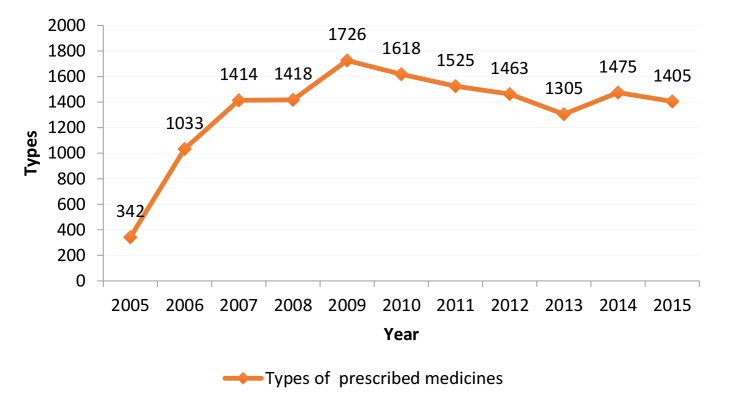
Types of Prescribed Medicines.

This indicator shows the quantity and variety of prescribed medicines, or in other words, the pharmacopoeia of physicians. The type of drug indicator shows the number of the drugs that doctors prescribe. It shows the medicines that are in the doctor’s pharmacopeia. A higher range of prescribed medicines included more effective medicines with no side effects indicates of better knowledge and expertise of physicians. The data showed that while prescribed medicines had the narrowest verity in 2005, it had the widest variety in 2009. However, since 2009, the indicator has decreased gradually (Figure 3). The experts considered initiatives adopted by MoHME aimed at removing some unnecessarily or harmful medicines from market as the reason for this issue: *“Since 2009, the MoHME has introduced some sorts of initiatives to control available medicines in the market, which has contributed to the reduction in prescription of medicines that were harmful, ineffective, or excessively used previously”* (KMO3).

Conversely, factors such as financial motivation and attempts made by physicians to have more clients led to decrease in the variety of prescribed medicines. : *“It is discussed that medicine is a business and in our society, patients expects doctors to treat them at the first visit... it is not possible to behave scientifically and start from the primary level, and in the case of lack of improvement, then refer patient to the secondary level and change drugs gradually. Physicians, at the first time, prescribe a strong medicine, due to patient request and in order to treat the patient rapidly, without considering possible side effects, [By following this approach, in the end, patient assumes the physician of high competence and skills]...”* (KMO2).

### Average Number of Medicines Per Prescription

This indicator shows the number of medicines prescribed per patient visit. The experts believed that the high values of indicator can make healthcare systems to spend excessively on pharmaceuticals and waste financial resources: *“Both MoHME and our organization (health insurance organizations) consider the decreased number of medicines [prescribed by doctors] as a positive point, while such a decrease is regarded as an unpleasant and negative condition by some patients. They think as ‘the more, the merrier’”* (HI2).

**Figure 4:**
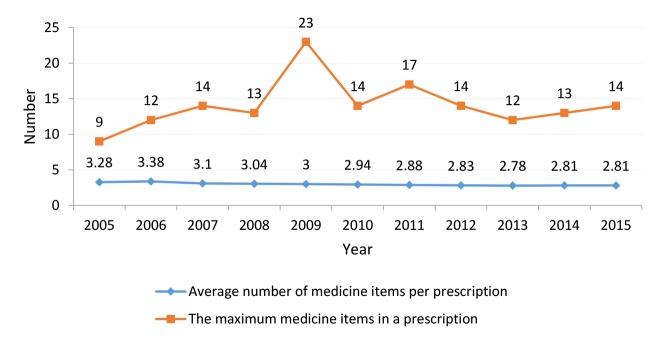
Average and Maximum of Medication Items Prescribd in Physicians’ Prescriptions.

Findings of this study revealed a gradual decrease in the average number of medicines per visit from 2005 to 2015 (Figure 4). The experts asserted that an increased sense of responsibility among physicians towards public health resulted in that they consider safety and suitability of drug while prescribing. Moreover, increased awareness of the patients about medicine overuse and side effects was proposed as another main reason for such a decrease: *“Fortunately, during recent years, responsible bodies have tried to promote the belief that excessive use of medications is not always good and can cause other problems. It seems that the beliefs have changed over time and this issue resulted in decreased demand for medicines”* (PH4).

However, factors such as writing more than one prescription per visit was identifies as another reason for such a decrease:* “One of our problems is that some physicians do not write all prescribed drugs in just one prescription. They know we (health insurance organizations) are monitoring their prescribed medicines. So they write medicines in two or more prescriptions separately”* (HI4).

As adopted initiatives by the MoHME has led to a decrease in the variety of medicines prescribed, these interventions have also had some impacts on the average numbers of medicines per visit and decreased it. Conversely, it is highly probable that factors such as increased burden of chronic diseases may have led to a relatively high increase in the indicator: *“Currently, the prevalence of chronic diseases such as Diabetes, metabolic syndrome, obesity, high blood pressure and so on is increasing, and sometimes when a physician visits a patient, he/she has to prescribe medicines for blood sugar, cholesterol and ..., these issues increase the average number of medicines prescribed per patient encounter as well”* (SP4).

According to the participants, entrance of newly graduated physicians to the job market could also increase the indicator: *“While reviewing [prescriptions] within the committee of prescription and consumption of medicines, we found out that older and more experienced physicians prescribe less drugs…; in contrast, recently graduated physicians prescribe more drugs with higher average of price”* (KMO1). Lack of competence in rational prescribing skills among recently graduated physicians was raised as the reason for this issue by most of the experts: *“We did not provide enough education on rational prescribing to medical students. I think if the principles of rational prescribing could be incorporated into their educational curriculums, many problems can be prevented or solved”* (KM2).

### The Maximum Number of Medicines Per Prescription

This indicator presents the maximum number of medicines prescribed by physicians within a certain period. It represents the highest number of drugs that doctors prescribed in their prescriptions. For example, if this indicator is 30, it means that in the year the largest number of drugs that are prescribed in a prescription is 30. Figure 4 shows the indicator’s trend between 2005 and 2015.

The maximum number of medicines in a prescription belonged to year 2009, in which there were prescriptions including 23 medications. Excluding 2009, an increasing trend was seen in the maximum number of medicines prescribed from 2005 to 2015. The experts asserted that the high number of medicines prescribed by newly graduated physicians was the reason behind such an increase. They pointed out that since 2009, when the number of recently graduated physicians increased in the province, there has been a gradual rise in the number of medicines per patient. In addition, migration of more experienced physicians from deprived provinces to better-off provinces can also be considered as another reason:* “The problem of the majority of the southern provinces of the country, eg, Kerman, is that these provinces are usually selected by young physicians to undertake their community services. These physicians pass the initial period of their practice in these provinces and after getting experience, they usually migrate to larger cities and metropolitan areas such as Tehran, Isfahan, Shiraz, or Mashhad; this issue brings about many challenges for these provinces”* (KMO 1).

Another factor that contributes to such an increasing trend is the shortage of combinatorial medicines and low diversity of medicines in the national pharmacopoeia*: “A problem which we have is that the number of those medicines that cover several health conditions simultaneously is scarce. In other words, due to lack of effective medicines, physician has to prescribe several medicines for each condition, which definitely increases the indicator”* (GP 1).

### Percentage of Prescriptions With More Than 4 Medicines

**Figure 5:**
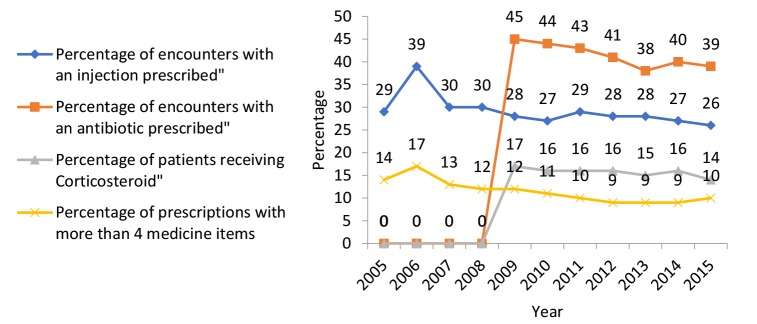
Percentage of Prescriptions With More Than 4 Medication Items, Percentage of Encounters With an Injection Prescribed, a Corticostroid Prescribed, an Antibiotic Prescribed.

The analysis of the data showed a decreasing trend in the percentage of prescriptions with more than 4 medications (Figure 5). Based on the experts’ opinion, the lower the indictor value, the more rational and appropriate prescriptions issued by physicians. A number of factors such as continued medical education (CME) programs designed by MoHME and universities of medical sciences to improve physicians’ competence in rational prescription were raised as contributors to the decreasing trend: *“CME courses held by universities attempted to make prescription more rational. The courses led to increased knowledge and skills [regarding rational prescription] among physicians as well as a decreased number of medication errors”* (R4)*.*

An increase in physicians’ awareness of drug interactions, which can be a result of the CME programs, was identified as an important factor in reducing the indicator*: “Fortunately, physicians now have more knowledge and awareness about the types of drugs available in the market and possible drug-drug interactions, and this was one of the reasons for this decrease”* (SP2).

### Percentage of Encounters With a Corticosteroid Prescribed

The analysis showed that during the recent years this indicator trend was fairly constant, showing a slight decrease (Figure 5). The increasing medical and pharmaceutical community’s awareness about the side effects of the excessive use of these medicines was considered as the reason for this trend by the experts: *“The Ministry of Health warned that by continuing the use of corticosteroid medicines, a disastrous rate of osteoporosis will soon show up among women taking these medicines. Besides, Kerman University of Medical Sciences [KUMS] has implemented several programs to inform people about the risks of these medicines, particularly Dexamethasone, which causes false obesity. I think these programs could gradually decrease the prescription of these medicines”* (PH2). Poor availability of proper alternative medicines in pharmacopeia and limited insurance coverage of alternative medicines were proposed as some reasons for high use of these medications. These factors encourage physicians to use more Corticosteroids: *“One of the reasons for the widespread use of Corticosteroids in Iran is unavailability of suitable analgesic medicines in pharmacopeia. Besides, the available [alternative] medicines are not fully covered by health insurance schemes. For this reason, some general practitioners use corticosteroids to decrease inflammation and pain in patients”* (KMO3).

### Percentage of Encounters With an Antibiotic Prescribed

The findings showed a decreasing trend in the percentage of visits with an antibiotic prescription over the recent years (Figure 5). Regulatory initiatives adopted by MoHME along with increased awareness of physicians concerning antimicrobial resistance, caused by overuse of antibiotics, were considered as the main reasons for the decreasing trend: *“The high use of these medicines causes drug resistance and when it (drug resistance) occurs we have to use stronger antibiotics; Stronger antibiotics are usually more expensive; Therefore, the average price of each prescription and the percentage of patients receiving injecting drugs will also increase...Thus if we continue this trend, we will enter into a vicious circle, which can have very dangerous consequences for public health. I think nowadays most of the physicians have accepted this issue”* (R5).

Nevertheless, there were some factors mentioned which in turn could increase the prescription of these medicines: *“It is likely that the low revenue of some physicians encourages them to prescribe medicines requested by patients in order to have more future patients... These physicians assume that they must prescribe medicines which decrease pain of patients and prevent future infections; this issue can increase the prescription of antibiotics”* (GP 1).

It should be mentioned that due to information deficiency, it was not possible to investigate the trend of percentage of visits with an antibiotic and corticosteroids prescribed from 2005 to 2008, but these indicators have gradually decreased since 2008 (Figure 4).

### Percentage of Encounters With an Injection Prescribed

The data revealed that this indicator had a relatively constant trend with a slight decrease over the study period (2005-2015) (Figure 5).

Participants believed that due to different reasons such as high costs, painful administration, and probability of infection, it is better to reduce the use of injections. In this regard, one of the specialists stated that:* “Generally, from a health system perspective, prescriptions including injecting medicines usually have high costs. From patients’ side, cost of injection in addition to the pain of injection and sometimes problems such as administration error or possible infection, encourage patients to lower the use of this form of medicines”* (PH1).

Some of the participants pointed out that the increased awareness of physicians about side effects of injecting medicines has led to a reduction in prescription of these medications in recent years: *“Currently, injecting medicines are not prescribed much; just special injecting antibiotics such as Penicillin are prescribed at low levels in medical offices, if a patient is too sick”* (GP 2).

Most participants acknowledged that lack of information and wrong beliefs among patients threaten adherence to rational prescription: *“There is a wrong culture among our people in which patients ask their physicians to prescribe certain medicines for them... this issue gets worse when our patients believe that injections are more powerful than oral medicines. Even some patients do not believe in a prescription in which there is no injecting medication and they do not adhere to it. It is also true about antibiotics, our people like antibiotics”* (SP1).

## Discussion

This study aimed to investigate rational use of medicines in Kerman province. To achieve this goal, eight main indicators of rational prescription were investigated over an 11-year period. The indicators investigated were as follows: average price of prescriptions, types of prescribed medicines, average number of medicines per prescription, maximum number of medicines per prescription, percentage of prescription with more than four medicines, percentage of encounters with a corticosteroid prescribed, percentage of encounters with an antibiotic prescribed, and percentage of encounters with an injection prescribed.

The findings showed that although the average number of medications per prescription has not increased much over the period, but the average price of prescriptions has increased. Increases in the price of law materials, devaluation of Iran’s national currency, and increases in the inflation rate (average 12% annually)^14^ were among the reasons proposed by experts for such a trend. However, when the indicators trend was compared with the average price of prescriptions for all Iranian physicians, the indicator amounted to US$3.67 in Kerman and to US$4.28 in the whole country.^8^ Experts believed that one of the most important reasons for this phenomenon was that physicians practicing in Kerman were much younger than the average of all Iranian physicians. A study conducted in India showed that despite adoption of the control measures by regulatory bodies, the average price of prescriptions was increasing. That study proposed to move towards greater use of generic drugs instead of brand drugs to control the increasing trend.^15^ In addition to irrational prescriptions that lead to increases in healthcare costs, evidence show that cost of drug production and supply, compared to other components of the health system, has witnessed a significant growth since 1990s. Factors such as increases in R&D expenditures within pharmaceutical companies and the emphasis on development of new drugs play major roles in the increasing trend. In addition to the costly process of new drug development, other factor such as effects of governmental policies on drug market or willingness of insurance companies to cover the newly developed medicines should be considered while investigating factors influencing costs.^16^

Our results confirmed the high degree of diversity in prescribed medicines by physicians in Kerman. However, in 2011, the indicator value was 1525 medicines in Kerman and 3490 at the national level.^8^ This difference indicates that limited range of medicines prescribed by Kerman physicians over the study period, which could result from limitations in the coverage of pharmaceutical drugs by health insurance schemes. In addition to factors like commercialization of medicines, regulatory initiatives taken up by MoHME that aim to omit harmful medicines from the market can be regarded as other influencing factors here. Researchers believe that to reduce antimicrobial resistance, rational and balanced use of various types of antimicrobials is much better than reduction in intake of one type of antibiotics.^17^

The average number of medicines per encounter had a gradual decreasing trend during the eleven years. This study showed that several factors such as increased sense of responsibility among physicians and increased awareness of patients regarding overuse of medicines had reduced the overuse of medicines in recent years. According to WHO and INRUD, the recommended value for this indicator ranges from 1.6 to 1.8 items.^9,18^ However, the minimum value of the indicator was reported to be 2.7 in the current study, ranging from 2.7 to 3.3. Interestingly, the indicator value in Kerman province was lower than the country average (approximately 3 medicines).^8^ Typically, the average value of this indicator fluctuates between 4.1 to 4.8 in developing countries and between 2.2 to 3.1 in developed countries.^11^ A study conducted in seven Southeast Asian countries reported that the average ranged from 1.4 to 3.8 medicines per encounter.^19^

The findings showed that the maximum number of medicines prescribed by Kerman physicians had increased over the study period. Experts believed that lack of knowledge among young physicians and lack of combinatory drugs in the pharmacopoeia were the main reasons for such an increase. The indicator value was 17 in Kerman and 31 in the country, indicating of a better situation for Kerman.^8^ A study conducted in Iran/Isfahan, reported that general physicians prescribed more number of medicines per patient as compared to specialists.^11^ Prescription of too many medications per encounter can be due to various reasons including, economic incentives, low knowledge about appropriate prescription, and lack of effective medicines.^18^

Percentage of prescriptions which had more than 4 medications showed a decreasing pattern in the current study, ranging from 9% to 17%. In 2011, this indicator was reported to be 10% for physicians practicing in Kerman province that was lower than the country average (15%) in the same year.^8^ Due to complications resulted from improper use of drugs, an international movement in different countries has been begun to restrict medicines overuse. Rational use of medicines can be improved with measures such as patient education, physicians and pharmacists’ education, and adoption of regulatory interventions.^20^ Surely, such measures can reduce the percentage of prescriptions with more than 4 items, improve the rational use, and reduce potential drug interactions.

The findings of this study indicated that factors such as increased awareness of healthcare providers about adverse effects of corticosteroids has led to the stabilization or even decrease in the prescription of such medicines. The indicator in Kerman province (9%-12%) was much lower than the country average (23%).^10^ Experts believed that efforts made by KUMS along with regulatory measures instituted by MoHME were effective in reducing the indicator.

The findings showed that similar to corticosteroid medicines, use of antimicrobial medicines has decreased over that period. This decreasing trend might be due to the regulatory measures taken up by MoHME, increased awareness of antibiotic resistance, and promotion of responsible usage of antibiotics by physicians. WHO has warned about antibiotic resistance that has resulted from overuse of antibiotics and called it as a threat to global public health that requires actions across all governmental and societal sectors.^21^

The ratio of antibiotics used per prescription, suggested by WHO, should not be more than 30% of all prescriptions.^9^ However, in the current study it ranged from 38% to 45%. In 2011, the ratio of antibiotics used per prescription was 45% in Kerman, which was equal to the country average in the same year.^10^ Prevalence of infectious diseases in underdeveloped and developing countries has led to high consumption of antimicrobial medicines in these countries. In this study, the proportion of antibiotics used was lower than that of other developing countries such as Sudan (71.8%), Ethiopia (58%), India (63.3%), and Nigeria (72.8%), and higher than some countries like Saudi Arabia (20%).^18,22-24^ A study conducted in Chinese hospitals to investigate prescription patterns reported that the average percentage of antibiotics usage was 29.90%, which was mostly attributed to regulatory mandates adopted by hospitals and the government.^9^

In our study, the average percentage of injection usage varied from 27% to 39%, which was slightly lower than the national average (41%).^10^ However, such a figure is far from the WHO standards (10%), which could indicate of a major deficiency of our health system.^9^ Factors such as increased awareness among physicians and high costs were raised by the experts as factors contributing to the observed decrease in the indicator. However, the experts believed that one of the main obstacles in rational use of injections was wrong beliefs among patients about the effectiveness of injection therapies and their irrational request for injection prescriptions. Another reason for irrational use of injection medicines was lack of coverage of effective medicines by basic health insurance that encouraged physicians to prescribe less effective drugs.^25,26^ In addition to high costs imposed to the health systems, irrational use of injectable drugs increases the incidence of infectious diseases, such as hepatitis and AIDS that incurs more costs to health systems in future.^5^ The value of this indicator in neighboring countries such as Bahrain, Kuwait and Saudi Arabia is 3.8%, 1.9% and 2%, respectively.^27-29^

The present study had some limitations that should be borne in mind when interpreting the results. One of the limitations was lack of inclusion of patients and their families, as one of the main stakeholders of pharmaceutical industry, into the study. However, due to lack of access to patients who could express their concerns, physicians were considered as the representatives of patients in the study.

The main strength of this study was the use of a mix-method approach to investigate the 11-year trend of prescription in Iran. Recruitment of variety of stakeholders and experts, ie, general practitioners, specialists, representatives of insurance organizations, MoHME and University officials, healthcare management and policy experts and researchers, who participated in the expert panel, was another strength of the study.

## Conclusion

This mixed method study investigated prescription indicators and trends among general practitioners and specialists in Kerman from 2005 to 2015. The findings indicated that these indicators were better in Kerman when compared to country averages. Experts cited several reasons for these differences. Initiatives adopted by Kerman University of Medical Sciences (KUMS), Kerman, Iran to provide training about rational prescription for physicians as CME programs as well as promotion of rational medication use among the public were considered as effective measures to control irrational medicine use. It should be noted that there is at least one university of medical sciences in all Iran’s provinces and they are responsible to provide healthcare in their catchment area. In fact, these universities should take a more active role in assessing and monitoring the extent of rational medicine use, comparing differences across health facilities, analyzing changes over time, and evaluating interventions.

## Ethical issues

The study was approved by the Ethics Committee at KUMS, Kerman, Iran. We reported all data as recorded in KUMS Medicine Database. Informed consent to audio record was obtained from each member of the expert panel. At the end of each expert panel session, minutes of the meeting were sent to participants to obtain their confirmation.

## Competing interests

Authors declare that they have no competing interests.

## Authors’ contributions

AM and SN were involved in the study design, data collection, data entry, analysis, and drafted the original manuscript. RD was involved in the study design, the statistical analysis, and drafting the manuscript. NH was involved in data collection and statistical analysis. AP was involved in study design, analysis and drafting the manuscript. JA was involved in drafting the manuscript.

## Authors’ affiliations

^1^Health Services Management Research Center, Institute for Futures Studies in Health, Kerman University of Medical Sciences, Kerman, Iran. ^2^Research Center for Modeling in Health, Institute for Futures Studies in Health, Kerman University of Medical Sciences, Kerman, Iran. ^3^Physiology Research Center, Kerman University of Medical Sciences, Kerman, Iran. ^4^National Institute of Health Research (NIHR), Tehran University of Medical Sciences, Tehran, Iran.

## Key messages

**Implications for policy makers**

Our mixed-method study showed that using experts’ opinion can improve interpretation of data on medication utilization. Furthermore, some interventions may promote rational use of medicines (The rational of medicines usage emphasizes on the rational prescription of drugs by practitioner and specialists and also rational use of prescribed drugs) as follows:

- Regulate drug market to replace non-effective or unsafe medicines with more effective medications and to include different medications’ types and forms,
- Providing physicians with feedback on their prescription patterns,
- Provide education on rational prescribing to medical students,
- Updating physicians’ knowledge on rational prescribing through continued medical education (CME) programs, and
- Providing educational massage on medication utilisation for population.

There are some factors that can be effective in drug trends that policy maker should consider, including:

- Economic conditions of the society and changes in the price of pharmaceutical drugs,
- Measures taken by the Ministry of Health and Medical Education (MoHME) in controlling and monitoring the drug market,
- Changing the pattern of diseases and increasing the burden of chronic diseases,
- Behaviours and motives of doctors, and
- Patient behaviours and thoughts.

**Implications for the public**

The rational of medicines usage emphasizes on the rational use of antibiotics, corticosteroids and injecting medications. The analysis shows that consumption of these medicines has decreased over an eleven-year period in Kerman province. It seems that initiatives adopted by Ministry of Health and Medical Education (MoHME) and providing physicians with continued medical education (CME) programs were among the main factors contributing to the reduction. In the time that it decreased in trends, mostly all the parameters were fixed except the MoHME intervention and experts believed that these interventions were effective.
